# Sequential dilatation of two balloons and double D-J stents for therapy of ureteroenteral anastomotic stricture in patients following radical cystectomy and Bricker urinary diversion

**DOI:** 10.4314/ahs.v25i1.26

**Published:** 2025-03

**Authors:** Ning Liu, Li Xing, Hong Chen, Shuqiu Chen, Menglan Li, Xiaowen Zhang

**Affiliations:** 1 SouthEast University Zhongda Hospital, Urology, Nanjing 210009, China; 2 SouthEast University Medical College, Nanjing 210009, China; 3 NHC Contraceptives Adverse Reaction Surveillance Center, Jiangsu Health Development Research Center, Nanjing 210036, China

**Keywords:** Bricker, Ureteroenteric anastomosis, Stricture, Dilatation

## Abstract

**Background:**

To determine the safety and efficacy of successive retrograde dilatation of two balloons and a double D-J stent for the treatment of ureteroenteral anastomotic strictures in patients who had undergone radical cystectomy and Bricker urinary diversion.

**Methodology:**

A total of 25 patients with ureteroenteral anastomotic stricture following radical cystectomy and Bricker urinary diversion were treated with sequential dilatation of two balloons (F18 and F24), while the remaining other 32 patients were only dilated once. All patients were treated with ureteroscope or flexible ureteroscope-guided retrograde implantation of twin D-J stents (F5).

**Results:**

Sequential dilation required significantly longer hospital stay and surgery time than single dilation. When sequential dilatation was compared to single dilatation, both the length of stay and the time it took to do the operation were clearly longer. After a follow-up of 6 to 24 months, the success rate of sequential dilatation was 61.5%, in comparison to 58.847.1% for single dilatation (P=0.83<0.05). All patients did not appear to have serious complications, such as hemorrhage, intestinal injury, or egression of the stent. No serious complications occurred in all patients, such as hemorrhage, intestinal injury, or egression of the stent.

**Conclusion:**

Sequential retrograde dilatation with two balloons and double D-J stents is thought to be safe and effective for uretero-intestinal anastomotic strictures in patients having with brick ureteral diversions, and it is associated with fewer sequelae. It is worthwhile for clinical purposes.

## Introduction

One of the most common consequences that can happen after a radical cystectomy with Bricker urinary diversion is ureteroenteral anastomotic stricture. The stricture rates range from 2 to 13%^1^-^3^. The aetiology is most likely the result of ischaemia at the anastomotic regionThe etiology is most likely to be ischemia of the anastomotic site^4^. Open surgery, with the highest success rate, is characterized by great difficulty, multiple complications and great damage to ureters and intestines. Although the success rate of open surgery is the highest, it is difficult, complicated, and more damaging to the ureter and intestines. As a result, Therefore, endoscopic treatment is still the firstline treatment, especially for patients with short stenosis or not suitable for open surgery. endoscopic treatment remains first-line therapy, particularly for patients with short stenosis or who are not candidates for open surgery. In this study, ureteroenteric anastomotic strictures were was treated using stageding,, sequential dilation of two balloons, and double D-J stents after a Bricker urinary diversion. The safety and effectiveness of this method are evaluated compared to conventional balloon dilatation., the safety and effectiveness of this method are evaluated.

## Methods

### Patients and methods

Between December 2012 and May 2019, 57 people patients who had received a the radical cystectomy and a Bricker urinary diversion for ureteroenteral anastomotic strictures were in treated by our department center between December 2012 and May 2019 were includedfor ureteroenteral anastomotic strictues. In these patients, 25 patients ones (26 ureters) were dilated with a balloon (COOK, F18), and another one (COOK, F24) in 4 weeks later. Double D-J stents (COOK, F5) were placed all the time and removed in 8-12 weeks afterin the second operation. The other remaining 32 patients (34 ureters) were dilated only once (COOK, F18) and placed with the same double D-J stents (COOK, F5) for 12 weeks.

### Clinical data

**Figure 1:**
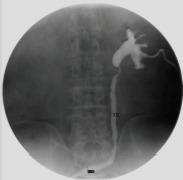
Anterograde angiography to assess hydronephrosis and the stenosis length

The main clinical manifestations were: include low back pain, repeated urinary tract infections, and fever. Nearly half of the patients had impaired renal function (serum creatinine >133 µmol/L). The period from postoperative to treatment was 6-30 months, and the left ureter stricture was the most common site of stricture. All patients underwent Computerized Tomography Urogram (CTU) or Intravenous Urogram (IVU) or anterograde angiography (Figure 1) to assess hydronephrosis and the stenosis length. See the following table for patient information (Table 1).

**Table 1:** Patient information

Parameter	Single dilatation	Sequential dilatation	P
Ureteral stricture(n)	32	32	
Left	20(62.5%)	15(60.0%))	0.85
Right	10(31.3%))	9(36.0%)	0.71
Both	2(6.2%)	1(4.0%)	0.70
Mean age (years)	66.4±8.0	65.9±5.9	0.83
Males (n)	25(78.1%))	21(84.0%)	0.57
Mean BMI (Kg/m2)	23.2±2.1	23.5±2.2	0.55
Median months to onset	12.9±14.9	13.2±4.6	0.86
Mean follow-up (months)	14.6±3.9	14.2±4.8	0.71
Mean length (cm)	1.2±4.2	1.1±4.1	0.99

### Surgical approach

**Figure 2:**
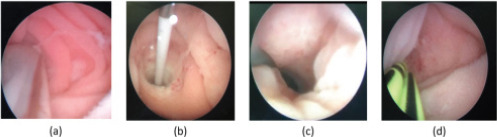
Surgical procedure: The balloon was inserted into the constricted ureter (a); The balloon was filled with saltwater at 20 atm for five minutes (b); Evident growth of the constraint (c); Placement of double D-J stents (COOK, F5) (d)

All patients were operated on under general anesthesia in a state of sterile urine. Preventive antibiotics were used before surgery. A ureteroscope or flexible ureteroscope was used to find locate the a ureterostoma in the ileal conduit. A superslip guidewire (Cook, 0.035″/145 cm) was introduced through the anastomosis anatomosis and verified by B-ultrasound to be placed in the afflicted renal pelvis. If the strictured ureterostoma was not foundnot able to be located precisely, an ileal conduit was inserted with a DSA-guided anterograde guidewire.. The balloon was pPlaced the balloon at least 5 cm into the strictured ureter under direct vision, keeping the pressure at 20 atm, and was removed it aftern 5 minutes. Double D-J (D-J) stents (COOK, F5) were placed after obvious expansion of the stricture, and the length of the stricture was recorded. No obvious bleeding or injury was observed in all cases (Figure 2).

### Follow-up survey

The follow-up time ranged from 6 to 24 months. The first time follow-up was arranged one month after the D-J stent waremoved, and patients were thereafter monitored every three months for up to two years. A computed tomography (CT) or ultrasonography was performed, and the serum creatinine level was reviewed. The disappearance of symptoms and hydronephrosis was considered a cure, and the improvement or disappearance of symptoms and the reduction of hydronephrosis was considered an improvement, both of which are collectively referred to as success. Failure occurred when the symptoms did not improve and the hydronephrosis did not decrease or worsen.

### Statistical analysis

Statistical Product and Service Solutions (SPSS) 26.0 (IBM, Armonk, NY, USA) was used to perform all statistical computations. The two-tailed Student's-t test (continuous variables) and Fisher's exact test were used for univariate statistical analysis (categorical variables). Statistical significance was assigned to tests with P<0.05.

## Results

The mean follow-up of the 25 patients (26 ureters) with in the sequential dilatation group was 14.2±4.8 months. Among these patients, 7 ureters were improved and, 9 were cured, and with the a success rate was of 61.5%. The average follow-up period for the othersof the 32 patients (34 ureters) in the single dilatation group was 14.6±3.9 months. In this group, Nine 9 ureters were improved, seven 7 were cured, and the success rate was 58.847.1%. The success rate was not statistically different, but increased numericallyin the sequential dilatation group was significantly higher than than in the single dilatation group (p<0.05, Table 2). There were significant differences in operation time and length of stay (Table 2). There was no hemorrhage, significant intestinal injury, or stent gression observed in any of the patients. All the failed patients chose renal fistula or long-term catheterization because they were unwilling to have receive replantation surgery. There was no increase in serum creatinine, even in patients with renal insufficiency.

**Table 2:** Comparison of patients undergoing different treatments

Patients	Operation time(min)	Length of stay(d)	Success rate (%)
Single dilatation	43.8±4.6	4.1±0.8	47.1%
Sequential dilatation	75.1±6.9	7.2±0.9	61.5%
P	<0.001	<0.001	<0.05

## Discussion

Two of the most popular surgical techniques for urine diversion are Bricker and Wallace. Although it has been reported that there is a significant difference in the incidence of anastomotic stenosis between the two operations (Bricker, 25.3% VS Wallace, 7.7%)^5^, Bricker is a simple and convenient operation, and Wallace has a higher risk of blockage due to recurrence at the ureteroenteric anastomosis or the chance of stones^6^. So in our department, Bricker is the mainstream. Ureteroenteric anastomotic stricture is associated with serious sequelae that lead to total or partial loss of kidney function, infetious complications, and the need for additional procedures. The most likely cause is ischemia in the anastomotic area^4^.

The traditional treatment for ureteroenteric anastomotic strictures is open surgery for reanastomosis, Schondorf^7^ reported that the long-term success rate is 91%. However, due to the adhesion formed during the previous operation, it is difficult and traumatic to have another open surgery, and the incidence of complications is high, which may easily lead to wound infection, vascular injury, and intestinal injury. Also, due to the effects of the initial operation, the majority of patients after a radical cystectomy are hesitant to have open surgery once more. Endourologic treatment, due to its simple operation, small trauma, and comparable short-term efficacy with open surgery^8^,^9^, has become another option for the clinical management of such complications quickly, including D-J stent or metal stent implantation, balloon dilatation, and stenosis incision.

Motoala et al.^10^ compared the efficacy of open surgery and balloon dilatation for ureteral stricture and concluded that dilatation should be the first option as long as the guidewire and catheter could pass through the stenosis segment. However, some recent literature has reported that the long-term success rate of balloon dilatation alone is very lowlower than open surgery. Van Son MJ Dimarco^11^ reported that the open surgery was superior to endourological methods (balloon dilatation included) in terms of patency duration. However, they also found a higher rate to compromise renal function in the open surgery group compared to the ballon dilatation group. long-term results of balloon dilatation alone in 52 cases with ureteroenteric anastomotic strictures, and found that the success rate was only 5% after 3 years of follow-up.

We improved the endourologic treatment. The fibrous scar in the stenosis segment was fractured first by balloon dilation, and then a larger diameter balloon dilation was used to completely fracture the fiber and its deep tissue in the same part, achieving the effect of “cold-knife incision” while avoiding complications such as hemorrhage and intestinal injury^12^. It was substantiated by ureteroscopy after dilatation. When compared to single balloon dilation, the success rate was clearly increased. Although sequential dilatation increased the operation time and length of stay, it still had significant advantages over open surgery^7^. In addition, no significant complications were found in the two groups of patients in this study, which proved that balloon dilatation was a safe and repeatable operation.

Double D-J stents were inserted into the ureter and arranged in parallel for at least 8 weeks. For good urine drainage, the space between the tubes must be big enough to allow for peritubular drainage instead of intraluminal drainage. At the same time, the double D-J stents can also play a good supprting and expanding role to reduce the occurrence of restenosis^13^. Operations were performed under the direct vision of a ureteroscope or flexible ureteroscope and assisted with B-ultrasound, which could ensure the balloon passed through the stricture smoothly and without damage. After dilatation, a ureteroscope or flexible ureteroscope could pass the stricture to observe the effect of dilatation, judge whether there was hemorrhage or intestinal injury, and measure the length of stenosis. Direct vision could avoid frequent fluoroscopy or angiography during the operation, and reduce the radiation damage to doctors and patients; In addition, the ileum bladder and ureterostoma it could be observed directly the ileum bladder and ureterostoma to prevent the omission of other conditions, such as tumor recurrence and stones.

This study does have some drawbacks. For starters, Firstly, this was a single-center research with a limited samples. Secondly, because of the intestinal folds and villi, it was sometimes difficult to identify the ureterostoma, and DSA was needed in this situation. Thirdly, the length of stay and operation time were increased, asell as hospitalization costs, in the sequential dilatation group. However, it was worthwhile for patients who had been effectively dilated for a long period of time.

## Conclusion

According to our preliminary clinical experience, results, sequential dilatation of two balloons and double D-J stents for the treatment of ureteroenteral anastomotic stricture in patients who have undergone radical cystectomy and Bricker urinary diversion is minimally invasive, reasonably safe, and effective. Long-term, extensive research should be undertaken to evaluate this strategy more thoroughly.

## Data Availability

The datasets used and analyzed during the current study are available from the corresponding author on reasonable request.

## References

[1] Anderson CB, Morgan TM, Kappa S, Moore D, Clark PE, Davis R (2013). Ureteroenteric anastomotic strictures after radical cystectomy-does operative approach matter?. J Urology.

[2] Ahmadi N, Ashrafi AN, Hartman N, Shakir A, Cacciamani GE, Freitas D (2019). Use of indocyanine green to minimise uretero-enteric strictures after robotic radical cystectomy. BJU Int.

[3] Reesink DJ, Gerritsen SL, Kelder H, van Melick HHE, Stijns PEF (2021). Evaluation of Ureteroenteric Anastomotic Strictures after the Intoduction of Robot-Assisted Radical Cystectomy with Intracorporeal Urinary Diversion: Results from a Large Tertiary Referral Center. J Urol.

[4] Richards KA, Cohn JA, Large MC, Bales GT, Smith ND, Steinberg GD (2015). The effect of length of ureteral resection on benign ureterointestinal stricture rate in ileal conduit or ileal neobladder urinary diversion following radical cystectomy. Urol Oncol-Semin Ori.

[5] Shah SH, Movassaghi K, Skinner D, Dalag L, Miranda G, Cai J (2015). Ureteroenteric Strictures After Open Radical Cystectomy and Urinary Diversion: The University of Southern California Experience. Urology.

[6] Lobo N, Dupre S, Sahai A, Thurairaja R, Khan MS (2016). Getting out of a tight spot: an overview of ureteroenteric anastomotic strictures. Nat Rev Urol.

[7] Christoph F, Herrmann F, Werthemann P, Janik T, Schostak M, Klopf C (2019). Ureteroenteric strictures: a single center experience comparing Bricker versus Wallace ureteroileal anastomosis in patients after urinary diversion for bladder cancer. Bmc Urol.

[8] Krafft U, Mahmoud O, Hess J, Radtke JP, Panic A, Püllen L (2022). Comparative analysis of Bricker versus Wallace ureteroenteric anastomosis and identification of predictors for postoperative ureteroenteric stricture. Langenbecks Arch Surg.

[9] Liu L, Chen M, Li Y, Wang L, Qi F, Dun J (2014). Technique selection of bricker or wallace ureteroileal anastomosis in ileal conduit urinary diversion: a strategy based on patient characteristics. Ann Surg Oncol.

[10] Schondorf D, Meierhans-Ruf S, Kiss B, Giannarini G, Thalmann GN, Studer UE (2013). Ureteroileal strictures after urinary diversion with an ileal segment-is there a place for endourological treatment at all?. J Urology.

[11] Alago WJ, Sofocleous CT, Covey AM, Thornton RH, Donat SM, Brody LA (2008). Placement of transileal conduit retrograde nephroureteral stents in patients with ureteral obstruction after cystectomy: technique and outcome. Am J Roentgenol.

[12] Zhang Z, Zhang C, Wu C, Yang B, Wang H, Hou J (2015). Progressive ureteral dilations and retrograde placement of single-j stent guided by flexible cystoscope for management of ureteroenteral anastomotic stricture in patients after radical cystectomy and bricker urinary diversion. J Endourol.

[13] Yagi S, Goto T, Kawamoto K, Hayami H, Matsushita S, Nakagawa M (2002). Long-term results of percutaneous balloon dilation for ureterointestinal anastomotic strictures. Int J Urol.

[14] Motola JA, Badlani GH, Smith AD (1993). Results of 212 consecutive endopyelotomies: an 8-year followup. J Urology.

[15] Van Son MJ, Lock MTWT, Peters M, van de Putte EEF, Meijer RP (2019). Treating benign ureteroenteric strictures: 27-year experience comparing endourological techniques with open surgical approach. World J Urol.

[16] DiMarco DS, LeRoy AJ, Thieling S, Bergstralh EJ, Segura JW (2001). Long-term results of treatment for ureteroenteric strictures. Urology.

[17] Poulakis V, Witzsch U, De Vries R, Becht E (2003). Cold-knife endoureterotomy for nonmalignant ureterointestinal anastomotic strictures. Urology.

[18] Wang Y, Ren X, Ji C, Zhong D, Wei X, Zhu Z (2023). A modified biodegradable mesh ureteral stent for treating ureteral stricture disease. Acta Biomater.

[R19] Ng CS, Streem SB (2004). Ureteropelvic junction obstruction: endourologic relocation of laterally inserting ureters. J Endourol.

